# Use of electromagnetic stimulation on an *Enterococcus faecalis* biofilm on root canal treated teeth in vitro

**DOI:** 10.1038/s41598-021-87922-4

**Published:** 2021-04-15

**Authors:** Beatriz H. D. Panariello, Justin K. Kindler, Kenneth J. Spolnik, Ygal Ehrlich, George J. Eckert, Simone Duarte

**Affiliations:** 1 Department of Cariology, Operative Dentistry and Dental Public Health, Indiana University School of Dentistry (IUSD), 1121 W Michigan St, DS406, Indianapolis, IN 46202 USA; 2 Department of Endodontics, Indiana University School of Dentistry (IUSD), Indianapolis, IN 46202 USA; 3 Department of Biostatistics, Indiana University School of Medicine, Indiana University School of Dentistry (IUSD), Indianapolis, IN 46202 USA

**Keywords:** Biofilms, Dental diseases

## Abstract

Root canal disinfection is of utmost importance in the success of the treatment, thus, a novel method for achieving root canal disinfection by electromagnetic waves, creating a synergistic reaction via electric and thermal energy, was created. To study electromagnetic stimulation (EMS) for the disinfection of root canal in vitro, single rooted teeth were instrumented with a 45.05 Wave One Gold reciprocating file. Specimens were sterilized and inoculated with *Enterococcus faecalis* ATCC 29,212, which grew for 15 days to form an established biofilm. Samples were treated with 6% sodium hypochlorite (NaOCl), 1.5% NaOCl 1.5% NaOCl with EMS, 0.9% saline with EMS or 0.9% saline. After treatments, the colony forming units (CFU) was determined. Data was analyzed by Wilcoxon Rank Sums Test (α = 0.05). One sample per group was scored and split for confocal laser scanning microscopy imaging. There was a significant effect with the use of NaOCl with or without EMS versus 0.9% saline with or without EMS (*p* = 0.012 and 0.003, respectively). CFUs were lower when using 0.9% saline with EMS versus 0.9% saline alone (*p* = 0.002). Confocal imaging confirmed CFU findings. EMS with saline has an antibiofilm effect against *E. faecalis* and can potentially be applied for endodontic disinfection.

## Introduction

The disinfection of the root canal system is of utmost importance in the success or failure of root canal treatment, though complete sterility is not possible^1^. Sodium hypochlorite is the time-tested gold standard of endodontic irrigation due to its nonspecific microbial killing as well as its ability to dissolve organic tissue^2,3^. The concentration of sodium hypochlorite which achieves the ideal balance of microbial killing and tissue dissolution while providing the lowest risk for cellular toxicity remains controversial. For standard nonsurgical root canal therapy, 6% sodium hypochlorite is commonly used as it is inexpensive and readily available^4^. In the case of an immature tooth undergoing regenerative treatment (REP), gentle irrigation with 1.5% sodium hypochlorite followed by gentle irrigation with 17% EDTA improves survival of stem cells of the apical papilla^5^. Indeed, 1.5% sodium hypochlorite is the irrigant of choice for REP as recommended by the American Association of Endodontists but must be followed by an intracanal medicament such as double antibiotic paste to achieve an acceptable level of disinfection, which requires multiple treatment appointments^6^. In either regenerative or traditional endodontic therapy, achieving an acceptable antimicrobial effect requires fresh sodium hypochlorite remain in the canal space for an extended time which is dependent upon the concentration^7–9^.

Sodium hypochlorite is toxic to virtually all human cell types and leaving it in the canal space during instrumentation increases the risk of apical extrusion. Apical extrusion of sodium hypochlorite can incite an intense inflammatory reaction resulting in long lasting swelling, bruising and severe pain for the patient. Presumably, a higher concentration would produce a more intense inflammatory response than a lower concentration if the same amount were extruded^10–12^. As of today, no other solution or material has supplanted the widespread use of sodium hypochlorite as a direct method of disinfection. However, much effort has been devoted to supplementing the action of sodium hypochlorite, such as passive ultrasonic irrigation and sonically activated irrigation; although both are widely used and highly successful in achieving acceptably disinfected canals in shorter periods of time, they both present an inherent risk for apical extrusion of irrigants and debris by their very nature^13–19^.

The International Society for Electromagnetic Dentistry (Tominaga Dental Clinic; Naruto, Japan) has developed a novel method for achieving root canal disinfection by energizing lower concentrations of sodium hypochlorite with electromagnetic waves, creating a synergistic reaction via electric and thermal energy^20^. An electromagnetic wave irradiation device attached to an active electrode (a specially coated ISO size 10 hand file) will create a circuit much in the same manner as an electronic apex locating device. Due to an insulating coating along the file (the active electrode), the electromagnetic waves are concentrated at the tip. The waves energize solutions through electric and thermal energy and has been coined electromagnetic stimulation (EMS) by its initial researchers. The research on EMS’s potential as an enhancing agent for root canal disinfection is very limited. So far, it has only been shown to be effective against planktonic bacteria^20–22^. Since endodontic pathogens exist as biofilms, which are up to 1000-fold more resistant to antimicrobials^23–25^, the existing literature on EMS is of little value in a clinical setting. In addition to the antimicrobial effect of EMS, there is potential for increased organic tissue dissolution, as well.

A fastidious pathogen found in many secondary and persistent endodontic infections^26–28^, *Enterococcus faecalis,* serves as an excellent model on which to test the antimicrobial efficacy of EMS. It is relatively easy to obtain, grow and maintain, and will form an established biofilm in a relatively short amount of time, on the scale of a couple weeks^29^. Although beyond the scope of the present study, the use of electromagnetic waves in routine endodontic treatment may prove to be of benefit in cases of apical periodontitis that present with a periapical radiolucency upon radiographic examination. Indeed, stimulation of osteoblasts as well as necessary growth factors for bone formation has been shown when EMS was applied to rat calvaria, resulting in increased bone healing^30,31^. With the potential for antimicrobial synergism, enhanced tissue dissolution, and more expedient bone healing, EMS has the potential to change the way current nonsurgical root canal treatment is performed.

Therefore, the aim of the current study is to evaluate the antibiofilm activity of EMS on a 15-day-old endodontic biofilm of *E. faecalis* formed on single rooted teeth. Samples were treated with 6% sodium hypochlorite (NaOCl), 1.5% NaOCl, 1.5% NaOCl with EMS, 0.9% saline with EMS, or 0.9% saline. After treatments, the colony forming units (CFU) was determined and confocal laser scanning microscopy imaging was performed. The null hypothesis is that root canaled teeth treated with EMS in combination with 1.5% sodium hypochlorite will not demonstrate a significant antibiofilm effect in comparison to those treated with 6% sodium hypochlorite alone. The alternative hypothesis is that root canaled teeth treated with EMS in combination with 1.5% sodium hypochlorite will demonstrate a significant antibiofilm effect in comparison to those treated with 6% sodium hypochlorite alone.

## Materials and methods

### Human tooth selection

**Figure 1:**
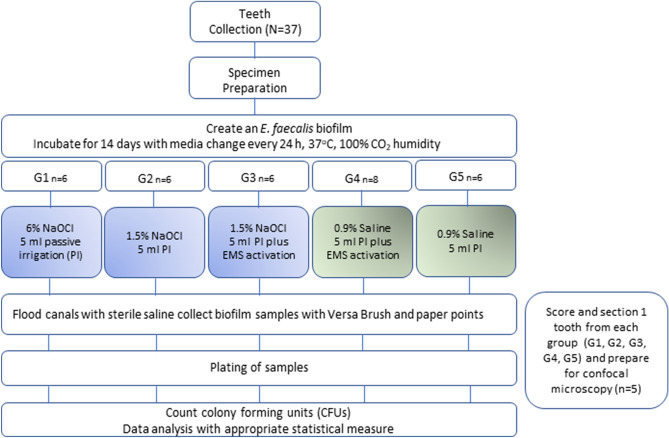
Experimental methodology.

An overview of the entire experimental methodology is provided in Fig. 1. Thirty-seven single rooted maxillary and mandibular human permanent teeth were collected and stored in a mixture of glycerin with 6% NaOCl. The human permanent teeth were not extracted for study reasons (Indiana University Human Subjects Office- study #1807417115). Only teeth with completely formed roots, free of decay, and at least 4 mm midroot diameter buccolingually or mesiodistally were included. Teeth exhibiting hypocalcification, restorations, decay, hypoplasia, fractures or cracks, incomplete radicular formation, fluorosis, and dentinogenesis or amelogenesis imperfecta were excluded. To determine whether teeth fit into these criteria, they were visually inspected.

### Root specimen preparation

The root canals of the tooth specimens were prepared by endodontic chemo-mechanical methods prior to inoculating the canal with *E. faecalis*. A diamond saw with water irrigation (Li’l Trimmer; Lapcraft; Powell, OH, USA) was used to cut off the crowns of the teeth. Root samples were prepared to a standard length of 12 mm. The canal spaces of the prepared root specimens were first negotiated with a K-file #10 (Dentsply Sirona; Tulsa, OK, USA) until its tip was visible at the apical foramen through magnification, followed by a #15 endodontic hand file (Dentsply Sirona, York; PA, USA) to length. This was followed by preparation with a size 25.07 Wave One Gold file (Dentsply Sirona; York, PA, USA) using a reciprocating motion in a Promark endodontic motor (Dentsply Sirona; York, PA, USA) and a second Wave One Gold reciprocating file, size 45.05, was used in the same manner and taken to full length to apical foramen, to standardize the apical foramen. During treatment, specimens were irrigated with 6% NaOCl (The Clorox Company; Oakland, CA, USA). Irrigation was performed with a 27Gx1-1/4″ (Covidien Monoject Endodontic needle; Walpole, MA, USA). Given that the selected needles have a 0.41 mm outer diameter and the apical foramen was prepared to 0.45 mm using a 45.05 Wave One Gold file, the chosen needle was small enough to reach within 1 mm of the apical foramen, as designed. The canal was patent and irrigation solution was able to exit the canal. Following preparation, the specimens were irrigated with 6% NaOCl and Ethylenediaminetetracetic Acid (EDTA) 17% (Henry Schein; Melville, NY, USA) for 3 min to eliminate the smear layer as described in the literature^32^. Teeth were stored in 6% NaOCl and glycerin at a ratio of 2:1 until ready for use, at which time they were autoclaved for 20 min at 121℃.

### Inoculation and biofilm formation

A solution of brain–heart infusion (BHI) broth (Acumedia; Lansing, MI, USA) was inoculated with a single colony of *E. faecalis* (ATCC 29,212) and incubated for 24 h (37℃, 5% CO_2_) to form the stock culture. The root specimens were coated with clarified and filter-sterilized pooled human whole saliva and prepared via 1 h 37℃ incubation^33^. Saliva was collected anonymously at the Oral Health Research Institute, Indiana University School of Dentistry, and was frozen until day of use (Indiana University Human Subjects Office, Office of Research Compliance – Indiana University study #:1406440799R002). To thaw, the saliva was placed in an incubator at 37℃ for one-hour and centrifuged for 10 min at 5000 rpm (Eppendorf 5804 R; Eppendorf Hauppauge, NY, USA). The supernatant was discarded, and the remaining liquid was sterilized by filtration through a 0.22 µm PES membrane filter (Genesee Scientifics; San Diego, CA, USA). The saliva-coated roots were placed in 24-well culture plates (1 sample per well) filled with 1.8 ml of sterile BHI and 0.2 ml of fresh 24 h stock inoculum and incubated at 37℃ and 5% CO_2_ for 14 days^34^. BHI solution was replaced every 24 h without the addition of new inoculum to prevent nutrient depletion.

### Experimental groups

After removal from the 24 well-plates, specimens were divided randomly into three experimental groups and two control groups with the number of specimens dependent on the disinfection protocol used (n = 8 in 0.9% Saline with EMS; n = 6 for all other groups). Samples were prepared for treatment by mounting in a sterilized sample cap 5A-1 (SKY-I, Japan) in fast-set alginate (Dentsply Sirona Jeltrate Plus; Dentsply Caulk, Milford, DE, USA). The alginate was supplied in individual use packets that were packaged in an aseptic environment. The experimental and control groups are depicted in Fig. 1.

### Electromagnetic stimulation experiments

**Figure 2:**
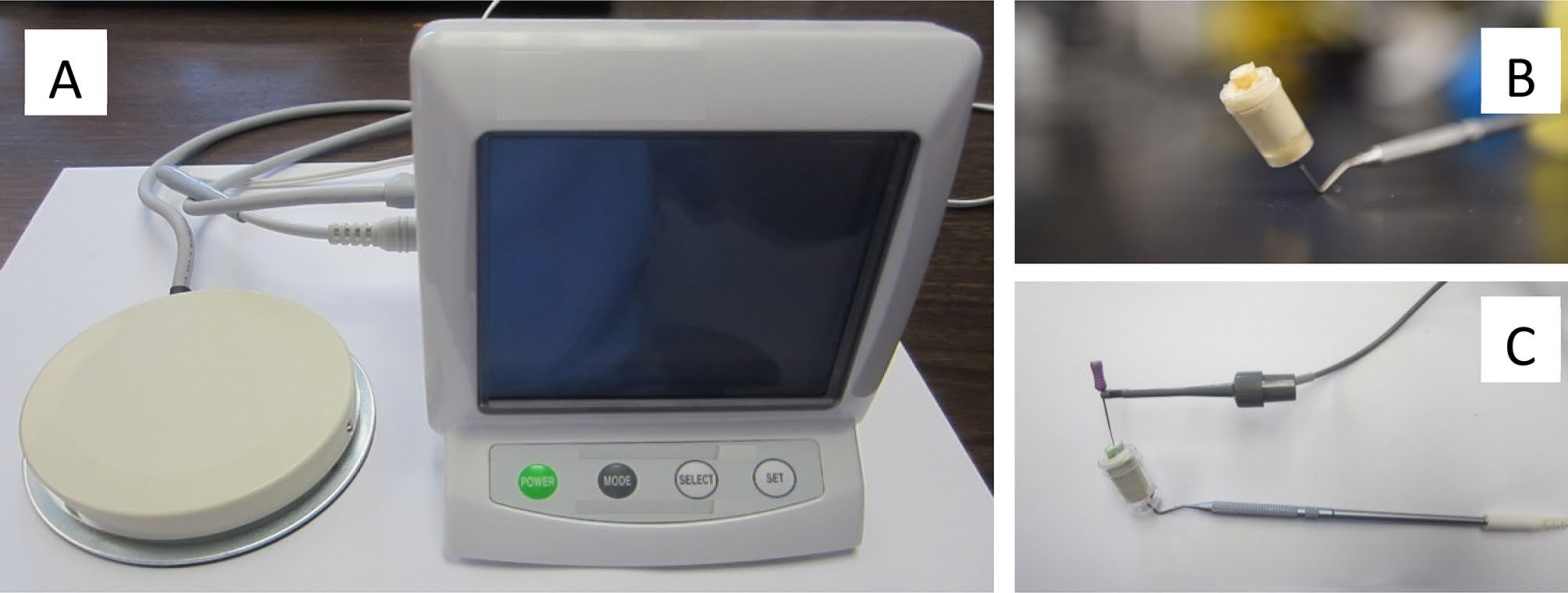
(**A**) represents the prototype used in the study. For circuit completion, a hole was bored in the inferior portion of the cap to allow the counter electrode, a modified periodontal probe, to be inserted (**B**). The specimen was mounted in alginate, leaving approximately 2 mm of tooth structure above the alginate (**B**). The active electrode was created by connecting an insulated #10 endodontic hand file to the device (**C**).

Figure 2 shows the high-frequency therapy prototype device (J. Morita MFG; Kyoto, Japan) used in the study. The device generates electromagnetic waves with a frequency 500–1000 kHz, and tone-burst waves with a large crest factor^20^. The manufacturer recommended settings were of 500 kHz, 80 mA and 70% duty. A specially coated ISO size 10 endodontic hand file (K-file No. 10; MANI, Utsunomiya, Japan) was used. The manufacturer supplied file is coated with parylene to insulate the file, and only the so last 2–3 mm of the file are exposed so that the electromagnetic burst only activates at the tip of the file. The 4–6 mm closest to the handle is also not insulated, to allow the active electrode to contact the metal file.

The EMS device (J. Morita MFG; Kyoto, Japan) requires a complete circuit to function. In a clinical scenario, a counter electrode is placed over the patient’s lip in the form of a shepherd hook. The active electrode is attached to an endodontic hand file via a clip. Teeth were mounted as previously described; for circuit completion, a hole was bored in the inferior portion of the cap to allow the counter electrode, a periodontal probe (YDM Corporation; Tokyo, Japan), to be inserted. This area was sealed using Revolution flowable composite (Kerr; Orange, CA, USA) and light cured for 20 s. The specimen was mounted in alginate, leaving approximately 2 mm of tooth structure above the alginate. The active electrode was created by connecting an insulated #10 endodontic hand file to the device. The parylene coating serves as an insulator, so the electromagnetic burst only activates at the last 2–3 mm of the hand file. The active electrode was inserted to a working length of 12 mm and activated for a 1 s burst at the manufacturer’s recommended setting of 500 kHz, 80 mA, 70% duty. A total of seven 1 s bursts were administered in the following manner: 3 bursts at the working length of 12 mm, 3 bursts at a working length of 9 mm, and 1 burst at a working length of 6 mm, as recommended by the manufacturer. The lengths were demarcated on the endodontic hand file with a marker to allow for expedient movement of the file during treatment. The one-second bursts were controlled by a rheostat, which is pressed each time a burst is desired. In the combination group, the canals were gently irrigated with either 1.5% NaOCl or saline with a 27-gauge needle up to 1 mm short of working length followed by immediate use of the EMS device. Gently irrigations describe moving the needle in an up and down motion, not fixed in one place while applying light pressure on the syringe, enabling only a small amount of fluid to exit the foramen. This method was chosen as it is how a patient would be treated in a clinical setting. All specimens were treated by the same calibrated operator. In the combination group, the canals were gently irrigated with either 1.5% NaOCl or saline with a 27-gauge needle up to 1 mm short of working length followed by immediate use of the EMS device. In the 1.5% NaOCl group, as well as the positive and negative control groups, the canals were gently irrigated with 5 ml volumes. Therefore, the irrigation protocol was as follows: the NaOCl or saline irrigant was inserted to 1 mm short of working length using a 27-gauge needle and 5 ml volume was gently expressed while using a gentle pumping motion, exactly as would be done by a clinician treating a patient.

### Assessment of antimicrobial activity

After treatment, coronal samples were immediately taken using a spiral utility brush (Versa Brush; Vista Dental Products, Racine, WI, USA) in a slow speed hand piece at 250 rpm for 1 min at a depth of 6 mm. Apical samples were taken by inserting a sterile size 30.04 paper point to a working length of 12 mm for 1 min because the Versa Brush did not reach the apical area. The same procedure was used for all groups. The spiral brush and paper point were transferred to 15 ml tubes (Falcon tubes; Thermo Fisher Scientific, Waltham, MA, USA) containing 5 ml of sterile saline. Biofilms were detached by sonication for 30 s then vortexed for 30 s. A ten-fold serial dilution was completed, followed by plating onto blood agar plates. After anaerobic incubation for 48 h in 5% CO_2_ at 37℃, colonies were counted, and CFUs/ml determined for statistical analysis.

### Scanning Confocal Electron Microscopy

In addition to the specimens in each of the 5 groups, one specimen was prepared and completed as described above in the 2 EMS experimental groups, as well as the two NaOCl and 0.9% saline groups, for a total of 5 teeth. Prior to sterilization, the teeth were scored longitudinally as described in a previous study^35^ using a straight handpiece with a diamond saw. This allowed separation of the specimen with a scalpel after treatment, exposing the root canal space for imaging. The canal space was stained with Live/Dead Bacterial Viability Kit (Baclight Bacterial Viability kit L7012; Thermo Fisher Scientific, Waltham, MA, USA). Three 0.5 mm stacks were taken starting from the apex and moving coronally for visualization of the treated biofilms at this portion of the tooth. A fourth 0.5 mm stack was taken individually at 6 mm from the apex to visualize a snapshot of the middle third of the tooth root.

### Statistical analysis

Due to a nonparametric distribution of data, CFUs were converted to log_10_. The effect of treatment group on log_10_ bacteria counts was made using Wilcoxon Rank Sum tests. A 5% significance level was used. The analyses were done in the software SPSS (IBM SPSS Statistics; version 21, Chicago, IL, USA).

### Ethical approval

All experiments and methods were performed in accordance with relevant guidelines and regulations. Human teeth collection (and relevant protocols) were approved by the Indiana University Human Subjects Office (study #1807417115) (Indianapolis, IN, USA). Saliva Collection [Protocol Title: Saliva Collection for In Vitro Studies (14-D-224)] was approved by the Indiana University Human Subjects Office, Office of Research Compliance – Indiana University (study #:1406440799R002).

### Informed consent

Informed consent was obtained from all teeth and saliva donors, all older than 18 years of age.

## Results

### Colony forming units

**Figure 3:**
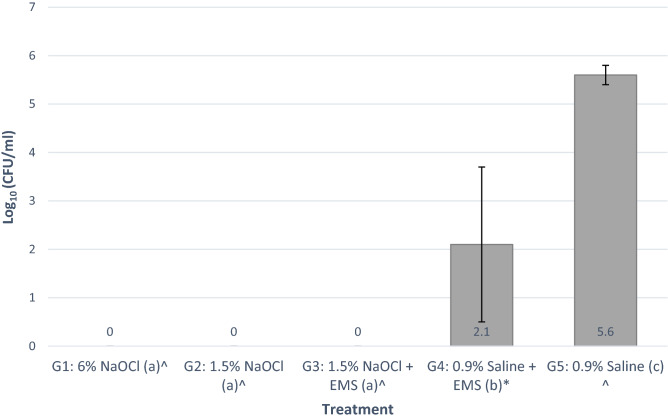
Average Log_10_ (CFU/ml) per group (^ indicates n = 6; * indicates n = 8; a different letter indicates that group was statistically significant from the other groups).

In all cases of disinfection with NaOCl, no colonies were formed after treatment. CFUs were counted in both the 0.9% saline and 0.9% saline with EMS groups. There was a significant effect with the use of NaOCl with or without EMS versus 0.9% saline with or without EMS (*p* = 0.012 and 0.003, respectively). EMS appeared to have an antibiofilm effect, however, as there were fewer CFUs formed when using 0.9% saline and EMS versus 0.9% saline alone (*p* = 0.002, Fig. 3).

### Scanning confocal electron microscopy

**Figure 4:**
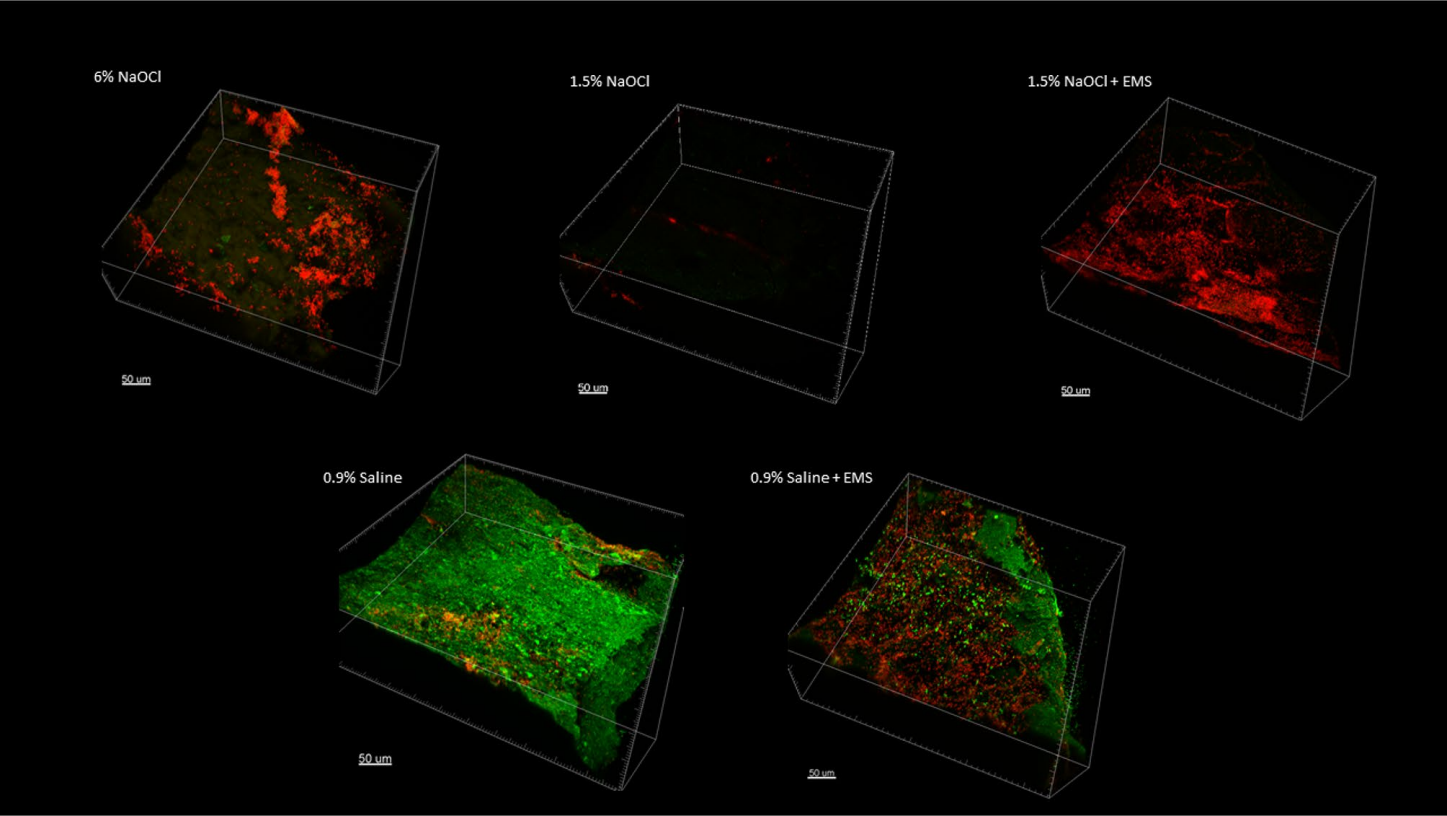
Apical 0–0.5 mm confocal images.

**Figure 5:**
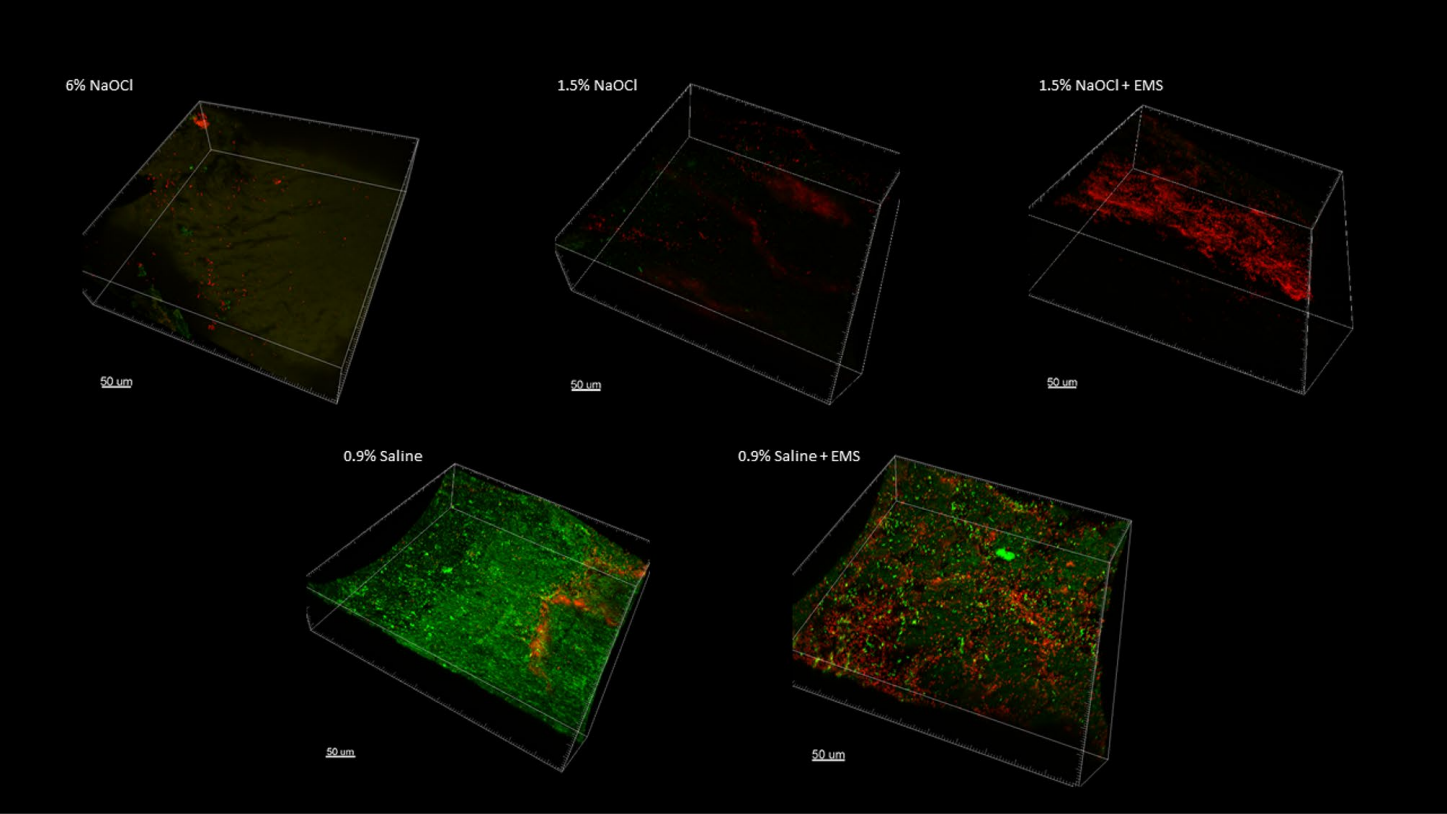
Apical 0.5–1.0 mm confocal images.

**Figure 6:**
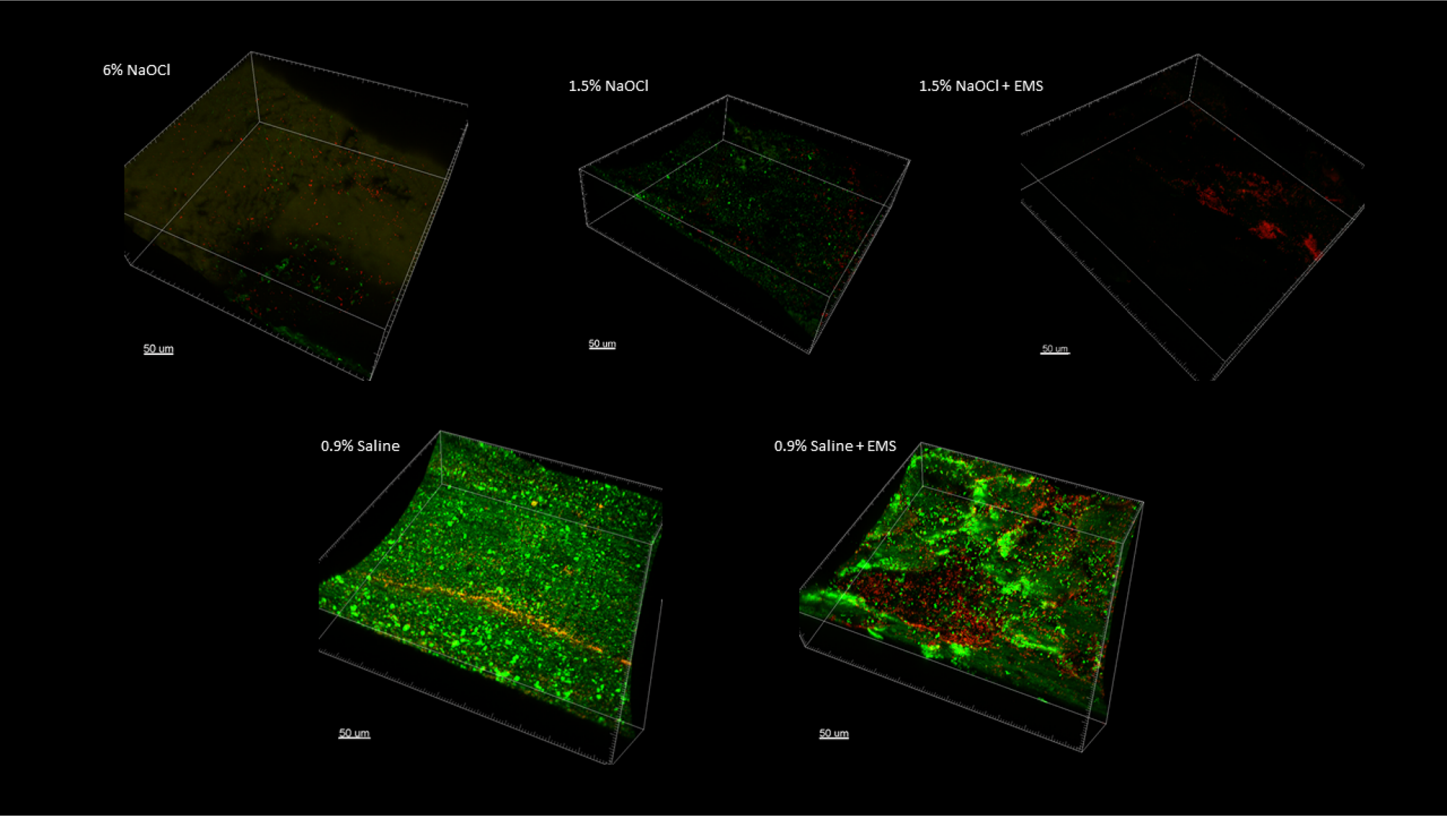
Apical 1.0–1.5 mm confocal images.

**Figure 7:**
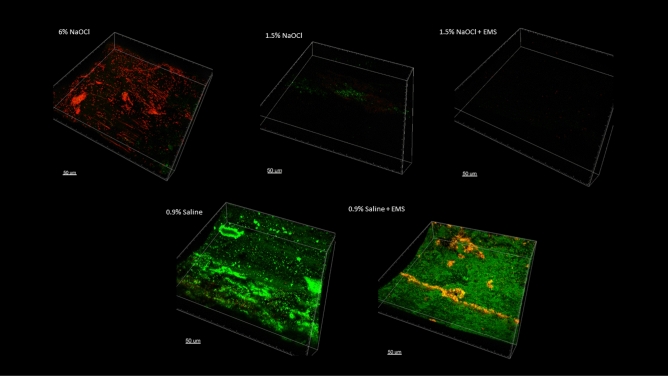
0.5 mm stack 6 mm coronal to the apex.

The apical 0.5 mm is presented in Fig. 4, the apical 0.5 to 1.0 mm is presented in Fig. 5, and the apical 1.0 to 1.5 mm is presented in Fig. 6. Figure 7 represents a 0.5 mm stack taken 6 mm coronal to the apex, for a snapshot into the middle third of the root canal space. In all instances in which NaOCl was used as an irrigant, confocal imaging shows complete eradication of the biofilm at the apical 1.5 mm, regardless of whether EMS was also used. When saline was used without EMS, the apical 1.5 mm contained a full thickness biofilm and nearly all cells were green, indicating no antibiofilm effect. When saline was used with EMS, there was a mixture of red cells, black space, and green cells, indicating some antibiofilm effect in the apical 1.5 mm.

## Discussion

Based on our results, the J. Morita prototype device can elucidate an antibiofilm effect against a 2-week-old biofilm of *E. faecalis.* This is evident as the CFU/ml counts in the saline with EMS group were less than half of what they were in the saline only group. This finding was corroborated with confocal imaging, where there were many more dead or missing cells in the saline with EMS group, whilst the saline only group showed a healthy, intact biofilm. Previous studies measuring the antibacterial effect of the J. Morita device used planktonic microorganisms^20^; to our knowledge, this is the first study that used a biofilm model. This initial study on the antimicrobial effectiveness of EMS will open a wide variety of research avenues and may eventually serve to maintain or enhance the current success rates of nonsurgical root canal therapy.

A synergistic reaction between the prototype device and NaOCl could not be determined because no colonies grew when root canals were irrigated with 1.5% or 6% NaOCl. Previous studies have shown eradication of an *E. faecalis* biofilm with as low as 0.000625% NaOCl in 1 min^36,37^. Other studies have found 2.5% NaOCl incapable of eradicating *E. faecalis* biofilms with as much as 40 min of contact time^37^. The combination of micro-electric assisted sonic agitation on 5.25% NaOCl was not capable to eradicate 21-day-old *E. faecalis* biofilms at 10 mA energy level in 60 s^38^. These differences are likely explained by study methodologies. In the present study, a higher energy level (80 mA) than in the previous study^38^ was used, moreover, a higher concentration of NaOCl (6% NaOCl) than in the previous study^38^ was also applied. It stands to reason, that with the high potential for shear forces in such straight and wide canals, as well as higher concentrations of NaOCl being used, even a 2-week-old biofilm would be eradicated.

The purpose of the confocal images was to provide a visual confirmation of what the CFU counts would tell about the antibiofilm effects of the various treatment modalities. Confocal imaging provides a three-dimensional image of a biofilm. For confocal imaging, groups are laid out by section. Live cells fluoresce a bright green color whereas damaged cells fluoresce a bright red color, which is a product of the molecules used for staining and imaging. During preparation, the samples are placed in several alternating washes of saline and stain solutions. This procedure can cause some dead cells to wash away, leaving black space. In all images in which NaOCl was used as the irrigant, there was either a mass of red cells, indicating cell death, or a large area or areas of black space, indicating removal of dead cells during the staining phase or during treatment with NaOCl. In addition to the effects seen with saline alone, we can see that the remaining cells in the 1.5% NaOCl + EMS samples fluoresced a very intense red, indicating a high PI to nucleic acid ratio. The cells in the 1.5% NaOCl group, however, do not appear and the ones that are fluoresced display a lower intensity of red. If we assume the 1.5% NaOCl samples washed out during treatment and not staining, this could indicate an enhancement of NaOCl (or one of its byproducts) uptake by outer membrane damage from the EMS treatment. However, this is difficult to confirm since both groups were irrigated with the same amount of NaOCl for the same amount of time prior to EMS treatment. One explanation could be that the author used a more forceful irrigation pattern in the 1.5% NaOCl only group as compared to the 1.5% NaOCl + EMS group, which is plausible, but unlikely. An alternative explanation is that NaOCl was highly effective in all scenarios and some damaged/dead cells were washed away during staining of certain samples.Confirmation of EMS’s enhancement of NaOCl could have been more easily attained had more cells remained viable in the 1.5% NaOCl group.

**Figure 8:**
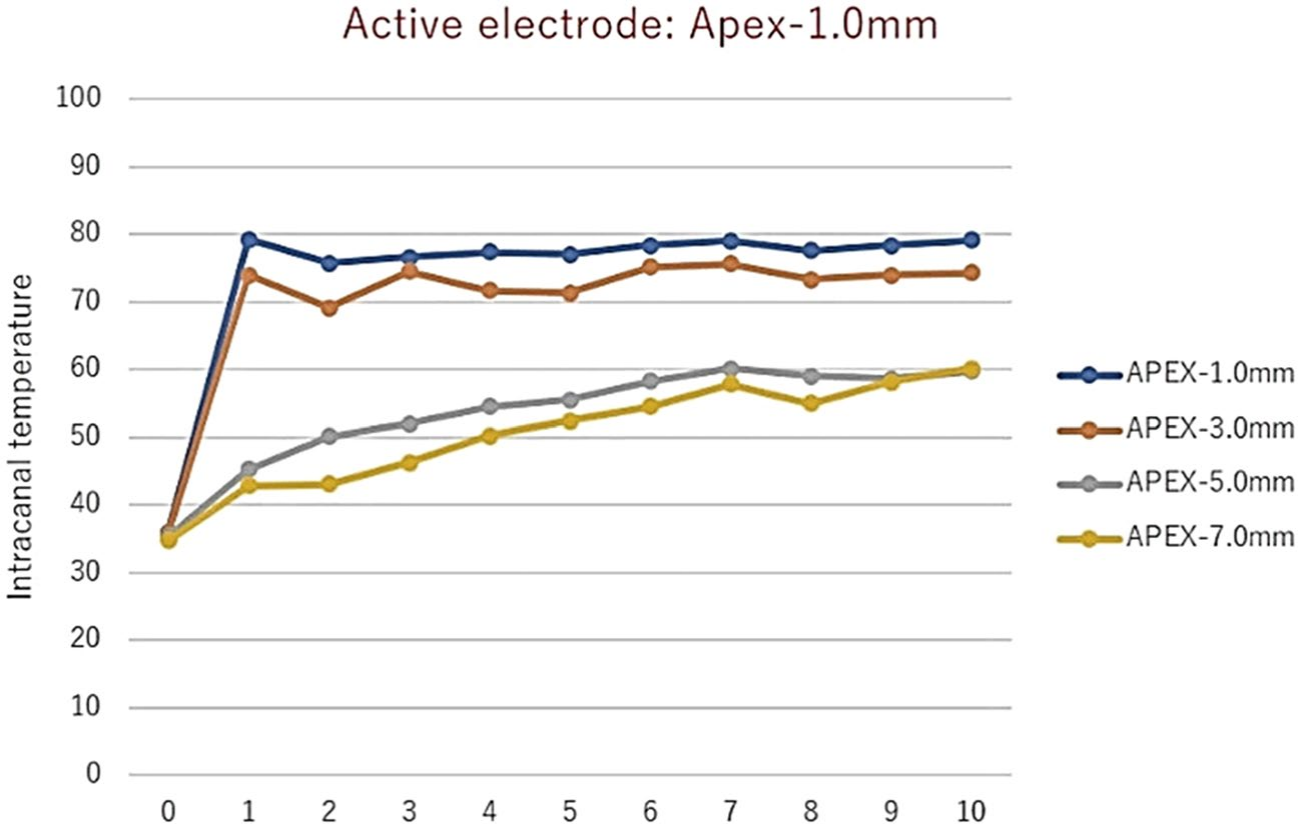
Intracanal temperature rises of mandibular incisors after 1 s EMS activation at various depths from the apex. NOTE: y-axis unit is degrees Celsius; x-axis unit is the number of times EMS activated for 1 s (Courtesy of Dr. Tominaga, International Society for Electromagnetic Dentistry).

The mechanism by which electric current exerts its effects on biofilms is currently unknown, but most theories involve increased uptake of antimicrobials by biofilm cells^39^. Since EMS exhibits an antibiofilm effect in 0.9% saline, perhaps its effect is due to a local generation of ions or oxygen, which disrupts or kills biofilm cells; however, such an explanation is purely speculation. Alternatively, a localized generation of heat could have been responsible for the effects seen. J. Morita theorizes that the antimicrobial capability of its prototype device is due to synergism between the electromagnetic pulses and any antimicrobial solution in the canal, which they have coined electromagnetic stimulation (EMS)^20–22^. It may be due to a localized increase in heat but there is no acoustic streaming or cavitation—only a short burst of electric current (1 s or less)^20,21^. In the in vitro study on planktonic bacteria, the solution’s temperature increased by 4–5℃ with each activation^20^. However, in unpublished data by the International Society for Electromagnetic Dentistry, intracanal temperature rose as much as 45℃ when EMS was used at 1 mm from the apex (Fig. 8); the rise was less dramatic farther back from working length likely due to an increase in canal diameter and the amount of solution present. The presence of more fluid would allow heat to dissipate, resulting in a smaller increase in heat. Standardized strains of *E. faecalis* have been shown to be susceptible to temperatures from 65 to 80 °C for 1–10 min, but clinical strains have also been shown to be resistant to 80℃ for as long as 3 min^40^.

The effects of micro-electric current on NaOCl’s tissue-dissolution abilities was compared with other activation methods, including sonic, ultrasonic, pipetting, and temperature^41^. It was reported that micro electrically activated 5.25% NaOCl has better results than 5.25% NaOCl without any activation on tissue dissolution efficiency^41^. It was also observed a positive combination of NaOCl activated with micro-electric current, heat, and agitation methods on NaOCl’s tissue-dissolving ability. This can be explained by the finding that when a micro electric current is activated NaOCl, the dynamic balance of the solution may change^41^. In addition, electrical activation of 1.25% NaOCl, 2.5% NaOCl and 5% NaOCl were previously studied in bovine muscle tissue. It was observed that higher concentrations of NaOCl and electrically activation considerably enhanced the efficacy of NaOCl. The effect of electrically activation on tissue dissolution was much greater than the same concentrations in the groups of NaOCl. It should be emphasized that lower concentrations of electrically activated NaOCl have a similar effect to more concentrated NaOCl solutions on tissue^42^. Future studies should consider utilizing lower concentrations of NaOCl, or perhaps using contact time rather than passive irrigation as the method for measuring solution delivery. For instance, rather than gentle irrigation with a set volume of irrigant, which will create shear forces, the canal could be filled with selected irrigant and allowed to sit for a predetermined amount of time before EMS activation takes place.

Not much is known about the effects that local electromagnetic current and its associated heat increase will have on host cells, such as osteoblasts, dental pulp stem cells, cells of the apical papilla, fibroblasts, periodontal ligament, etc. In rat calvaria, upregulation of osteoblast proliferation as well as an increase in growth factors necessary for bone mineralization were noted^31^. Clinically, periapical lesions in bone were found to heal up to 4 times faster when treated with the prototype device as compared to control^22^. Moreover, EMS would be used on anesthetized patients as it is being used during an endodontic procedure, so perception of electric current is unlikely. In addition, it has already been used clinically in Japan with no reported adverse effects^21,22^. Follow-up studies should therefore examine these effects on other cell lines such as stem cells and cells of the periodontal ligament.

In a study examining the effects of tobramycin on a *Pseudomonas aeruginosa* biofilm, the authors applied a 2-mA current to the biofilm in the presence of tobramycin and found a significant increase in bacterial killing over biofilms injected with oxygen and tobramycin or a control in which tobramycin was used alone. The bioelectric effect could not be explained away by a change in pH, temperature increase, or disruption of the biofilm extracellular matrix by the addition of gas^43^. In regenerative endodontic procedures (REPs), the most used antibiotics are TAP or DAP, which contain ciprofloxacin, metronidazole, and/or clindamycin or minocycline, depending on the clinical use or clinician’s preference^44^. Given the increase in bactericidal activity seen in the previously mentioned study on *P. aeruginosa*, follow up studies using EMS with TAP or DAP could be conducted to determine if there are potential uses in REPs or if these antibacterial are sufficient for use in non-surgical root canal therapy.

Based on the confocal images obtained, the present insulation design may result in a limited zone of effect with EMS. At the apex and at 6 mm back from the apex, two locations directly affected by activation, more bacterial killing is visualized in the form of red cells or black space than at 1 mm or 1.5 mm coronal to the apex. Follow-up studies should modify file insulation design to see if the EMS effect can be spread more evenly throughout the canal. For instance, horizontal slits could be placed in the insulation material every 1–2 mm to increase the area affected. This may also result in less need for multiple activations. Finally, certain in vivo characteristics may affect the current flow of EMS, such as dentin thickness, canal diameter, and amount of solution present. All these variables must remain standardized to determine the actual effects of EMS, so clinical results may differ from what is found during a laboratory experiment.

The findings of this study indicate the use of EMS with saline has an antibiofilm effect against *E. faecalis* when compared with irrigation with saline alone. This effect was not as potent as irrigation with 6% NaOCl. Furthermore, since there was no bacterial growth in all groups in which NaOCl was used, a synergistic effect could not be determined. Therefore, the null hypothesis that EMS used with 1.5% NaOCl would have an antibiofilm effect like irrigation with 6% NaOCl alone cannot be rejected. A limitation of this study is that this is an in vitro study. However, in vitro studies are essential to observe the results of the therapies before they can be translated to in vivo or clinical studies. At present, the most applicable clinical use for EMS may be its ability to expedite bone healing as already proven via clinical studies.

We conclude that EMS is effective for the disinfection of root canal in vitro. Follow-up studies should focus on utilizing lower concentrations of NaOCl, consider other disinfectants such as CHX, TAP or DAP, modify file insulation designs, or examine the effects on stem cells, osteoblasts, or cells of the periodontal ligament. Subsequently, the treatment proposed in this study can be translated to future clinical studies, including the test of lower concentrations of NaOCl in combination with EMS.
